# A Chemoattractant Role for NT-3 in Proprioceptive Axon Guidance

**DOI:** 10.1371/journal.pbio.0020403

**Published:** 2004-11-23

**Authors:** Barış Genç, P. Hande Özdinler, April E Mendoza, Reha S Erzurumlu

**Affiliations:** **1**Department of Cell Biology and Anatomy, Louisiana State University Health Sciences CenterNew Orleans, LouisianaUnited States of America

## Abstract

Neurotrophin-3 (NT-3) is required for proprioceptive neuron survival. Deletion of the proapoptotic gene *Bax* in *NT-3* knockout mice rescues these neurons and allows for examination of their axon growth in the absence of NT-3 signaling. TrkC-positive peripheral and central axons from dorsal root ganglia follow proper trajectories and arrive in close proximity to their targets but fail to innervate them. Peripherally, muscle spindles are absent and TrkC-positive axons do not enter their target muscles. Centrally, proprioceptive axons branch in ectopic regions of the spinal cord, even crossing the midline. In vitro assays reveal chemoattractant effects of NT-3 on dorsal root ganglion axons. Our results show that survival factor NT-3 acts as a short-distance axon guidance molecule for muscle sensory afferents as they approach their proper targets.

## Abstract 2

In vivo and in vitro experiments suggest a role for NT-3 in guiding axons from the spinal cord to their muscle targets

## Introduction

Neurotrophin-3 (NT-3) is a key requirement for the development of proprioceptive inputs to motor neurons (Chen and Frank 1999; Chen et al. 2003). Mice deficient in NT-3, its tyrosine kinase receptor, TrkC, or in TrkC-positive neuron-specific transcription factor Runx3 display severe ataxia associated with the absence of muscle spindles, and loss of proprioceptive neurons in dorsal root ganglia (DRGs) or their axons (Ernfors et al. 1994; Klein et al. 1994; Tessarollo et al. 1994; Fariñas et al. 1996; Liebl et al. 1997; Inoue et al. 2002; Levanon et al. 2002). NT-3 is expressed in the ventral spinal cord, in the developing limb buds, and in intrafusal bag fibers of muscle spindles later in development (Copray and Brouwer 1994; Fariñas et al. 1996; Tojo et al. 1996). When sensory axons contact developing myotubes, they induce muscle spindle differentiation, forming ring-like spiral nerve endings around them. In the chicken embryo, limb ablations or anti-NT-3 antibody injections into limb buds lead to elimination of TrkC-positive neurons and decreased innervation of motor neurons (Oakley et al. 1995, 1997). Is NT-3 only a chemotrophic survival factor for muscle sensory afferents, or does it have additional roles in the development of the proprioceptors and the establishment of the monosynaptic reflex arc? Here we provide evidence that NT-3 acts as a chemoattractant for sensory axons during the final phase of their target-directed pathfinding.

## Results

### TrkC-Positive DRG Neurons Are Rescued in *Bax/NT-3* Double Knockout Mice

**Figure 1:**
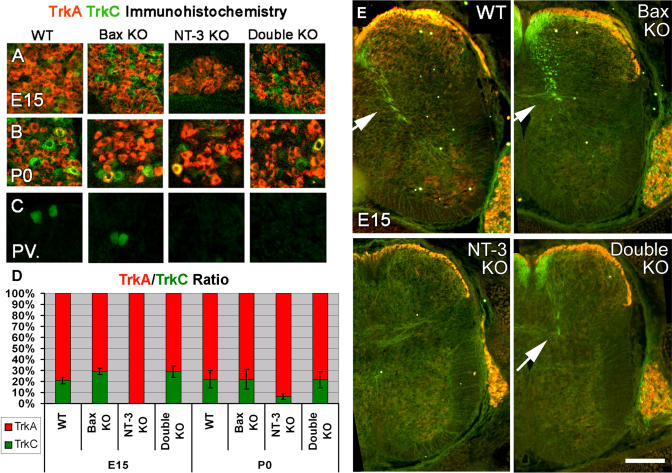
TrkA/TrkC and PV Immunohistochemistry in DRG and Spinal Cord Red represents TrkA, green represents TrkC (and PV in [C]), and yellow represents co-expression. (A) TrkA/TrkC immunostaining in E15 DRG. TrkC-positive neurons normally eliminated in *NT-3* KOs are rescued in double KOs. (B) TrkA/TrkC immunostaining at P0 in DRG. (C) PV immunostaining in P0 DRG. Rescued TrkC-positive cells fail to express PV. (D) Ratio of TrkA-immunopositive cells to TrkC-immunopositive cells in E15 and P0 DRGs. Data are presented as percentage of cells with standard deviation. Double KOs always had similar ratios to *Bax* KOs, and *NT-3* KOs had the least amount of TrkC-positive cells, if any. (E) TrkA/TrkC immunostaining in E15 spinal cord. Arrow points to group Ia fibers. Dorsal is up. Scale bar: 50 μm (A–C), 1 mm (E).

Mice lacking proapoptotic protein Bax allow for distinguishing survival effects of neurotrophins from other effects. Bax-deficient sensory neurons no longer require neurotrophins for survival (White et al. 1998; Patel et al. 2000), thus they can be used to examine axonal effects. We bred *NT-3* heterozygote and *Bax* knockout (KO) mice to obtain mice with double KO of both *NT-3* and *Bax* genes, and examined proprioceptive axonal projections. All *NT-3* and double KOs died within 48 h after birth (Tessarollo et al. 1994). We performed TrkA/TrkC double immunohistochemistry (Huang et al. 1999), enabling detection of both proteins in the same sample. TrkC-positive cells (Figure 1A) and fibers (Figure 1E) were absent in *NT-3* KOs at embryonic day (E) 15. Two subsets of DRG cells expressing either TrkA or TrkC were detected in double KOs, similar to wild-type (WT) or *Bax* KO animals. Surprisingly, at postnatal day (P) 0, a few cells expressed TrkC even in *NT-3* KO animals in the *Bax* ^+/+^ genetic background, and some cells co-expressed TrkA and TrkC, regardless of the genotype (Figure 1B). In order to quantify our results, we analyzed the ratio of TrkA and TrkC immunopositive cells from four different DRGs of animals of different genotypes. At all ages studied, Bax/NT-3 double null DRGs had TrkA/TrkC ratios similar to those of Bax null DRGs, and higher than those of NT-3 null DRGs (Figure 1D). TrkC-positive neurons rescued by *Bax* deletion, however, failed to differentiate properly, as evidenced by the lack of expression of the proprioceptive molecular marker Parvalbumin (PV) (Figure 1C).

### NT-3 Is Necessary for Proper Innervation of Motor Neurons

**Figure 2:**
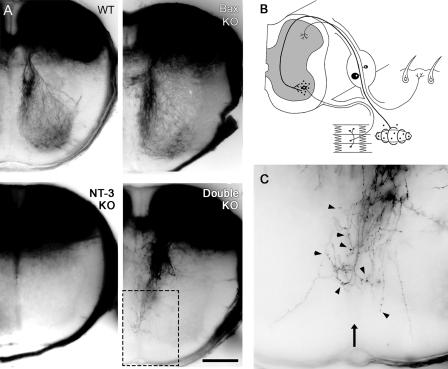
Axonal Projections in the Spinal Cord after DiI Labeling of DRG at P0 (A) Rescued DRG proprioceptive neurons fail to properly innervate motor neurons in double KOs. Instead, some axons are directed towards the ventral midline; they cross the midline and branch. (B) Schematic drawing of the monosynaptic reflex arc as it normally develops. Small black dots represent NT-3 released centrally by the motor neurons and peripherally by the muscle spindles. (C) High-power magnification of the inset in (A). Arrow points to the midline, and arrowheads point to synaptic bouton-like structures. Scale bar: 1 mm (A), 400 μm (C).

**Figure 3:**
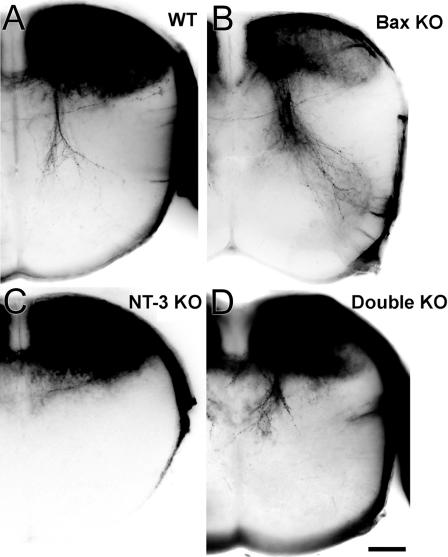
Sensory Axons Labeled with DiI through the DRG at E17 (A) DiI-labeled fibers in WT spinal cord. Notice proprioceptive axons extending towards the motor neurons located in the ventral horn of the spinal cord in cross section. (B) Bax null spinal cord. (C) NT-3 null spinal cord. Stained fibers are restricted to the nociceptive axons in the dorsal horn, as evidenced by the complete absence of labeling in the ventral spinal cord. (D) Bax/NT-3 double null spinal cord. Although fibers extend into the ventral spinal cord, they never grow towards the motor neurons, but are directed towards the midline instead. Scale bar: 100 μm.

TrkA/TrkC-positive fibers in the spinal cord could be detected at E15 (Figure 1E). TrkA-positive fibers were restricted to and terminated in the dorsolateral spinal cord, whereas TrkC-positive fibers entered the cord dorsomedially, and descended into the ventral horns in WT (Ozaki and Snider 1997) and *Bax* KO embryos. There was no detectable TrkC expression in *NT-3* KO spinal cord, indicating complete absence of proprioceptive fibers. In double KO spinal cord, TrkC-positive fibers entered the dorsal spinal cord and descended medially in a manner similar to that seen in WT or *Bax* KO cases. However, it was not possible to follow TrkC immunolabeled fibers all the way to their terminal zones in any of the cases. Next we examined the central projections of DRG axons with the lipophilic tracer DiI at P0. In WT and *Bax* KO pups, proprioceptive afferents entered the dorsal spinal cord and followed a medial course towards the ventral horn. They then turned laterally towards motor neurons in the lateral motor column, where they branched and terminated (Figure 2A). DiI labeling was confined to dorsal spinal cord in *NT-3* KOs (Figure 2A), as reported earlier, consistent with a complete absence of proprioceptive innervation (Ernfors et al. 1994; Tessarollo et al. 1994). In double KOs, proprioceptive afferents initially followed a trajectory similar to that of WT counterparts, but most of them failed to project all the way to the ventral cord and into the lateral motor column. Instead, they arborized near the ventral midline; some crossed the midline and extended into the contralateral ventral cord (Figure 2A and 2C). In order to distinguish between a role for NT-3 in initiation of motor neuron innervation and a role for maintenance, we repeated the DiI labeling at E17. Innervation patterns of E17 spinal cords (Figure 3) were similar to those at P0 (Figure 2). Dorsal horns of all genotypes were filled with DiI-labeled fibers corresponding to nerve growth factor–dependent nociceptive axons. In WT and *Bax* KO embryos, proprioceptive fibers extended towards ventral horn motor neurons (Figure 3A and 3B), whereas the ventral horns of the *NT-3* KO embryos were devoid of innervation (Figure 3C). In the *Bax/NT-3* double KOs, DiI-labeled fibers entered the ventral spinal cord, but extended towards the midline instead of the ventral horn (Figure 3D), in a pattern similar to that observed at P0. Our data point to a complete absence of proprioceptive innervation of the ventral horn of the Bax/NT-3 null spinal cord throughout the developmental stages investigated. As the sensory axons never reach motor neuron dendrites in the ventral horn (Figure S1), the stretch reflex arc circuit is not established. The failure to initiate contact between sensory axons and motor neurons in the absence of NT-3 suggests a requirement for NT-3 for proper axon targeting in addition to a role in sensory axon maintenance.

### NT-3 Is Required for Proper Peripheral Innervation

**Figure 4:**
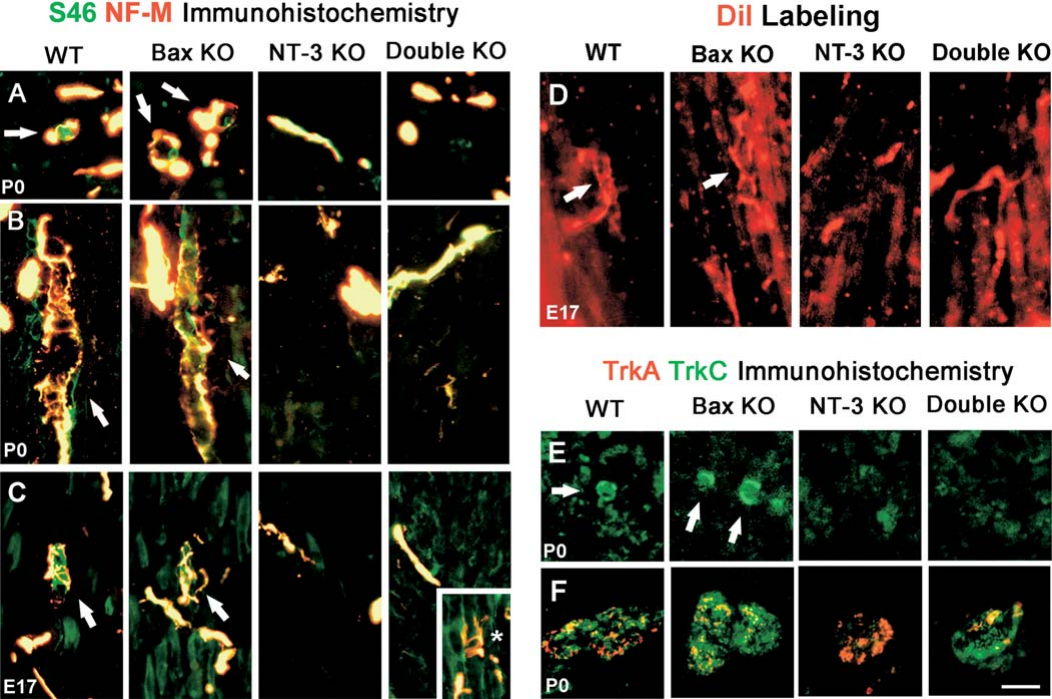
Muscle Spindles in Gastrocnemius Muscle and TrkA/TrkC Staining in the Tibial Nerve at P0 (A) NF-M (red) and S46 (green) immunostaining in cross section of gastrocnemius muscle at P0. There are no muscle spindles detected in double KOs. (B) NF-M (red) and S46 (green) immunostaining in parallel sections of gastrocnemius muscle at P0. (C) NF-M (red) and S46 (green) immunostaining in parallel sections of gastrocnemius muscle at E17. Double null muscles are mostly devoid of muscle spindles, except for one spindle-like structure detected (shown in inset, denoted by the asterisk). (D) Muscle spindles detected by DiI labeling through the DRG. Gastrocnemius muscle is sectioned at 40 μm thickness in parallel plane to the muscle fibers. (E) Muscle spindles detected by TrkC staining in cross section of gastrocnemius muscle at P0. (F) TrkA (red) and TrkC (green) immunostaining in the tibial nerve cross section at P0. TrkC-positive fibers are missing in *NT-3* KOs. Red-green overlap (yellow) is due to the thickness of the section and overlapping of red- and green-labeled (TrkA and TrkC) fibers present at different focal depths, rather than co-localization. Arrows indicate muscle spindles. Scale bar: 50 μm (A, B, D), 25 μm (C, E), 75 μm (F).

In order to study peripheral innervation in double KO animals, we investigated spindle development in the gastrocnemius muscle. Muscle spindles could be identified easily in P0 WT and *Bax* KO animals by their characteristic morphology (Figure 4A and 4B). Proprioceptive fibers labeled with neurofilament-M (NF-M) antibody formed ring-like spiral endings wrapped around intrafusal bag fibers labeled with S46 antibody, specific for slow developmental myosin heavy chain protein (Miller et al. 1985). As reported earlier, there were no muscle spindles in *NT-3* KO animals (Ernfors et al. 1994). On the other hand, *Bax* KO animals had more spindles than WT, and spindles were in clusters similar to animals over-expressing NT-3 in the muscle (Wright et al. 1997). Although NT-3-dependent cells were rescued, no muscle spindles were detected in the limbs of double KOs (Figure 4A and 4B). Muscle spindles could be observed with TrkC antibody in WT and *Bax* KO animals, but not in *NT-3* or double KOs (Figure 4E). In both *NT-3* and double KO animals, NF-M-labeled fibers could be detected in muscles, thus the muscles of these animals were not completely devoid of nerve fibers. Since there were no TrkC-positive fibers in these muscles, we think that these NF-M-labeled fibers correspond to motor axons. Absence of muscle spindles might be due to a failure in initiation of differentiation by proprioceptive axons, or a failure of maturation and maintenance in the absence of NT-3. To distinguish between these two possibilities, we investigated muscle spindle development at E15 and E17 with S46/NF-M immunostaining as well as DiI labeling. No structures with the characteristic muscle spindle shape could be detected in any of the genotypes at E15 (Figure S2). However, numerous developing spindles could be identified at E17 in WT and *Bax* KOs, but not in *NT-3* KO embryos (Figure 4C). Bax/NT-3 double null muscles were devoid of spindles (Figure 4C), except for one spindle-like structure observed in one leg of an embryo (Figure 4C, inset, denoted by the asterisk). DiI labeling through the DRGs at E17 yielded similar results, with muscle spindles identified in parallel sections in WT and Bax null muscles (Figure 4D), but not in *NT-3* or double KO animals. Although DiI-labeled fibers could be detected in double null muscles, they never formed ring-like structures characteristic of muscle spindles. We also examined TrkA/TrkC expression at P0 in the tibial nerve, which carries sensory fibers to the gastrocnemius muscle as well as the skin of the lower leg. In *NT-3* KOs, TrkA-positive axons could be seen in the tibial nerve but there were no TrkC-positive axons; in contrast, TrkA- and TrkC-labeled axons are both present in WT, *Bax* KO, and double KO animals (Figure 4F). These results suggest that although proprioceptive axons follow proper trajectories in distal peripheral nerves, they fail to innervate their target muscles in the absence of NT-3.

### NT-3 Is a Chemoattractant for DRG Axons In Vitro

**Figure 5:**
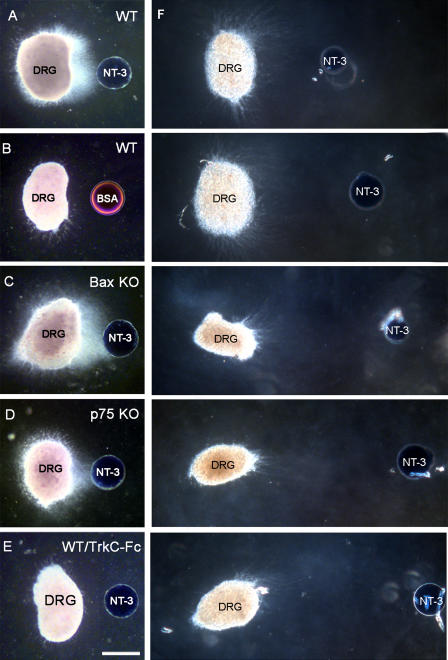
Chemoattraction of E13 DRG Axons to Local NT-3 Observed by In Vitro Co-Culture Assays (A) WT DRG with NT-3-loaded bead. (B) WT DRG with BSA-loaded bead. (C) Bax null DRG with NT-3-loaded bead. (D) p75 null DRG with NT-3-loaded bead. (E) WT DRG with NT-3-loaded bead and TrkC-Fc in the medium. (F) WT DRG with NT-3 loaded beads placed at increasing distances away from the ganglia (range, 500–1,200 μm). Scale bar: 150 μm (A–E), 350 μm (F).

**Figure 6:**
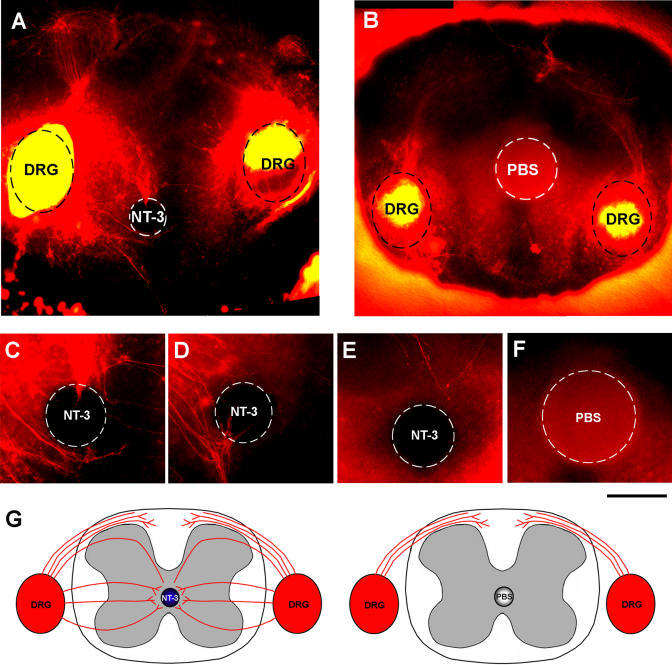
Chemoattraction Towards NT-3 Beads Placed in E13 Spinal Cord DRG Explant Co-Cultures (A) NT-3 bead placed in the midline of E13 WT spinal cord. Notice axons labeled through the DRGs (circled with black dashed lines) growing towards the bead (circled with white dashed lines) enter the spinal cord at ectopic loci instead of dorsal spinal cord. (B) PBS-loaded bead in E13 spinal cord. All labeled axons extend along the dorsal spinal cord, where they terminate. (C) High-power image of the bead in (A). Notice labeled axons surrounding the bead. (D) High-power image of an NT-3-loaded bead. Notice axons bundled around the bead. (E) High-power image of an NT-3-loaded bead. Notice the axons approaching the bead via the dorsal spinal cord. (F) High-power image of a PBS-loaded bead. No labeled fibers were observed around control beads. (G) Summary of our observations from E13 spinal cord DRG organotypic cultures. In control cultures fibers extend along the dorsal spinal cord, where they normally enter the gray matter at E13. In the presence of an ectopic NT-3 source localized at the midline, these axons grow towards the NT-3 bead. NT-3 also initiates axon growth from the DRGs, entering the spinal cord at ectopic lateral loci, growing towards the bead, surrounding the bead, forming nerve bundles, and branching around it. Scale bar, 175 μm (A and B), 100 μm (C–F).

To test the hypothesis that NT-3 acts as a chemoattractant for sensory axons, we performed a series of in vitro assays. Proprioceptive axons in mice enter the gray matter in the spinal cord and advance ventrally parallel to the midline by E13 and reach the motor neurons by E15 (Ozaki and Snider 1994). We co-cultured collagen-embedded E13 WT DRG explants with NT-3-soaked sepharose beads (*n* = 26). Control cultures were set up using bovine serum albumin (BSA)– or phosphate buffer saline (PBS)–soaked beads (*n* = 12). DRG axons began extending towards the localized NT-3 source by the end of the first day and consistently displayed a strong chemoattraction by 3 d in vitro, whereas they did not show such preference for BSA-loaded control beads (Figure 5A and 5B). This attraction was not due to survival support of NT-3 because Bax null ganglia displayed the same chemoattraction (Figure 5C; *n =* 16). NT-3 may act through either TrkC, or the p75^NTR^. We repeated the co-culture experiments with DRG explants derived from *p75^NTR^* KO mice (*n =* 18). Axons of these ganglia also showed strong chemoattraction towards the NT-3 beads (Figure 5D). Finally, we used diffusible TrkC receptors conjugated to IgG constant regions (TrkC-Fc) added to the medium (*n =* 6) to deplete soluble NT-3 from the collagen gels (Figure 5E). In the presence of TrkC-Fc the chemoattraction was completely blocked, demonstrating that the effect we see is specific for NT-3. In order to investigate the active range of our beads, we have repeated the cultures with WT E13 DRGs by placing the beads at increasing distances from the ganglia (*n =* 4 each) (Figure 5F). There was still preferred growth towards the bead at 1,200μm, the longest distance studied, although the number of axons and the extent of growth were not as robust. Next, we set up E13 DRG spinal cord explant cultures using NT-3-loaded beads placed at the midline at mid-spinal cord level as an ectopic NT-3 source (*n =* 15). Control cultures were set using PBS-loaded beads (*n =* 6). DiI labeling through the DRGs revealed numerous fibers entering the spinal cord at ectopic regions and growing towards the NT-3 beads (Figure 6A), surrounding the beads and forming bundles around them (Figure 6C–6E). In control cultures, all labeled fibers were directed towards the dorsal spinal cord and terminated there, where they normally enter the gray matter at E13 (Figure 6B). No axons were observed around the PBS-loaded control beads (Figure 6F). Axons that normally enter the gray matter through dorsal spinal cord grow towards the midline when presented with a localized NT-3 source, and new axon growth towards the NT-3 bead is initiated from DRGs, entering the spinal cord at ectopic loci at lateral mid-spinal cord (Figure 6G). NT-3 is therefore capable of acting as a chemoattractant for DRG axons.

## Discussion

Relatively few studies have implicated NT-3 as a chemoattractant agent for sensory and motor axons. Previously noted chemoattractant action of the embryonic mouse maxillary process on trigeminal ganglion neurons (Lumsden and Davies 1986) is now attributed to NT-3 and brain-derived neurotrophic factor (BDNF) (O'Connor et al. 1999). Recently, Tucker et al. (2001) showed that developing sensory and motor axons in limb slice cultures preferentially grow towards neurotrophin-soaked beads instead of following their normal trajectories. Conversely, beads soaked with neurotrophin function-blocking antibodies led to reduction of sensory and motor axon growth towards the limb. In transgenic mice, which over-express NT-3 under the *nestin* promoter in the central nervous system, the course of the proprioceptive afferents are altered and directed towards the regions with high levels of ectopic NT-3 expression in the spinal cord (Ringstedt et al. 1997). Ringstedt et al. considered the possibility that NT-3 may play a chemoattractant role during the innervation of ventral horns by proprioceptive afferents. However, earlier findings of normal proprioceptive afferent trajectories in chicken embryos despite injection of function-blocking NT-3 antibody into the spinal cord (Oakley et al. 1995) led them to discount this possibility. In that study, though, assays on the effectiveness of antibody perturbation on NT-3-dependent cell survival showed that effectiveness was significant (approximately 90%) but not complete (Oakley et al. 1995). Ringstedt et al. (1997) argue that while central sensory axons may still navigate properly in the absence of NT-3, ectopic expression of NT-3 disrupts their targeting. Recently, another member of the neurotrophin family, BDNF, has been suggested to act as a chemoattractant for sensory axons innervating ear (Tessarollo et al. 2004). In a gene replacement strategy in which BDNF expression was driven by the NT-3 promoter, vestibular axons rerouted towards ectopic sources of BDNF in the cochlea that normally expressed NT-3 and were not innervated by these axons. Ectopic NT-3 supplied using osmotic pumps and adenovirus-mediated expression induce sensory axon growth during regeneration (Zhang et al. 1998, Bradbury et al. 1999, Oudega et al. 1999, Ramer et al. 2002), and induce axonal plasticity of corticospinal axons in injured adult spinal cord (Zhou et al. 2003), where the sprouting axons from the intact site cross the midline towards the NT-3 source on the lesion side of the spinal cord. Our present results are in agreement with these observations, and provide further evidence that NT-3 acts as a chemoattractant for sensory afferents.

Previously, normal motor neuron innervation was rescued in *NT-3* KO animals by over-expressing NT-3 selectively in their muscles (Wright et al. 1997). It appears that peripheral NT-3 alone is sufficient for rescuing proprioceptive neurons in *NT-3* KO animals and proper axonal pathfinding in the spinal cord. However, it is unclear whether NT-3 is absent from the ventral spinal cords of these animals. The possibility has been raised that motor neurons may retrogradely transport NT-3 in muscle to the spinal cord (Chen and Frank 1999). Assays to determine whether any NT-3 is present in the ventral horns of these mice would be informative.

Recently, Patel et al. (2003) reported their observations on *Bax/NT-3* double KO mice they bred. Their results are significantly different from ours. They see no proprioceptive afferents in the periphery of the double knockouts and note that central proprioceptive afferents terminate in the intermediate spinal cord without extending ventrally. Their observations are based on PV immunostaining and DiI labeling of the peripheral nerves. We noted that PV immunolabeling is diminished in our *Bax/NT-3* double KOs. In various manipulations of the neurotrophins it has been noted that molecular markers for proprioceptive axons such as PV or calcitonin gene-related peptide for nerve growth factor–responsive axons are compromised (Ringstedt et al. 1997; Patel et al. 2000), thus PV immunostaining in *Bax/NT-3* double KOs cannot reveal the extent of proprioceptive axons in the periphery.

Seventy-three percent of retrogradely labeled gastrocnemius muscle afferents were reported to be expressing TrkC RNA in the adult rat DRG, although some of these (about 10%) may represent cutaneous innervation (McMahon et al. 1994). Presently, it is not clear whether TrkC protein is also made by cutaneous afferents and if so at what stage in development this receptor is expressed by cutaneous axons. Based on the available evidence showing that all proprioceptive neurons are eliminated in NT-3 or TrkC null mice, we think that TrkC staining in the tibial nerve mostly represents proprioceptive axons in the vicinity of their peripheral target. Altogether, our findings suggest a role for NT-3 in initiation of muscle innervation and spindle differentiation by the proprioceptive axons.

Patel et al. (2003) examined DiI-labeled central DRG axons in the spinal cord of three E17 and two P0 cases, and report that central proprioceptive axons stop in the intermediate laminae, never entering the ventral cord. We have examined nine *Bax/NT-3* double KO cases, and often incomplete DiI labeling gives the impression that there are no axons reaching the ventral spinal cord. We have also seen cases similar to theirs (*n =* 4), but at higher magnification these axons did not have terminal boutons and were not completely labeled. However, with complete fills (*n =* 5), it was possible to trace these axons into the ventral midline and across to the contralateral side, and visualize terminal boutons at their tips (see Figure 3C). Previously, Arber et al. (2000) reported that members of the Ets family of transcription factors, Er81 and Pea3, are expressed by DRG neurons as well as motor neurons and their target muscle fibers. They found that in the spinal cord of *Er81* KO mice, ventral projections of proprioceptive axons were mostly absent, and very few axons made it to the ventral cord. Patel et al. (2003) note that the phenotype they observed in their *Bax/NT-3* double KO mice is quite similar to that of *Er81* KO mice. They provide evidence that NT-3 induces Er81 expression in DRG explants in vitro. Patel et al. (2003) report that Er81 mRNA expression is diminished (but not abolished) in both *NT-3* KO and *Bax/NT-3* double KO mice, while their immunohistochemistry shows much less protein expression in the double KO mice. It is highly possible that a small but considerable number of DRG cells express the transcription factor Er81 and that their axons grow beyond the intermediate levels of the spinal cord in *Bax/NT-3* double KO mice. While the phenotype of *Er81* KO mice is quite dramatic, and most proprioceptive axons stop within the intermediate spinal cord, it is important to note that a few axons still find their way to the ventral spinal cord and target properly to the motor neurons (Arber et al. 2000).

Patel et al. (2003) also present observations from islet2^DTA^ mice, which lack a significant portion of the motor neurons in the ventral cord. In these mice PV immunostaining shows axons in the ventral horns. Patel et al. argue that since motor neurons are absent in these mice, NT-3 secreted by them could not be a signal for proprioceptive axons to enter the lateral motor columns. However, there is no evidence showing that NT-3 mRNA or protein expressed in the ventral spinal cord is exclusively from motor neurons, and there are no available data indicating that in islet2^DTA^ mice, NT-3 expression in the ventral spinal cord is abolished (Yang et al. 2001; Pun et al. 2002). Studies in embryonic mice reported NT-3 mRNA in the ventral horns of the spinal cord, but it is not definitive that both mRNA and protein are expressed solely by motor neurons. In the adult spinal cord, while motor neurons express high levels of NT-3, other cells, including glia, also express it (Zhou and Rush 1994; Dreyfus et al. 1999; Buck et al. 2000). Our present results, along with those from transgenic mice with NT-3 over-expression in ectopic regions of the spinal cord (Ringstedt et al. 1997), argue for a role of NT-3 in chemoattractant axon guidance of proprioceptive axons in the spinal cord.

Finally, in culture assays, we see a strong chemoattraction of DRG neurons to localized sources of NT-3. This response is seen in WT, Bax null and in p75 null DRG explants, and in the absence of any other neurotrophins or target-derived axon guidance molecules. Furthermore, in vitro, sensory axon response to NT-3 does not appear to be dose-dependent (Ringstedt et al. 1997; Tucker et al. 2001). NT-3 is capable of attracting axons along distances of up to 1 mm in collagen gel matrix, covering the physiological range it needs to attract axons during development. Previous studies with exogenous or local applications of NT-3 to developing primary sensory axons have indicated that this neurotrophin can attract and induce axonal branching (Ulupınar et al. 2000; Özdinler and Erzurumlu 2001, Özdinler et al. 2004).

Along the monosynaptic stretch reflex pathway, only Wnt-3 has been implicated as an axon arborization factor in the spinal cord (Krylova et al. 2002). Another molecule, Slit2, expressed in the midline and by motor neurons (Wang et al. 1999) is capable of inducing axonal branching (Nguyen Ba-Charvet et al. 2001; Özdinler and Erzurumlu 2002). DRG neurons express Robo receptors, which bind to Slits, and proprioceptive axons are therefore capable of responding to Slit signals (Wang et al. 1999). Slit2 does not cause repulsion of NT-3-responsive DRG axons in vitro (Nguyen Ba-Charvet et al. 2001), but causes ectopic branching and arborization of trigeminal axons in the brainstem (Özdinler and Erzurumlu 2002). Thus, Slit2 might also be involved in terminal branching of propioceptive axons in the ventral cord and in the midline branching observed in our double KOs.

Lack of PV expression in *Bax/NT-3* KO mice suggests that PV expression might be responsible for proper axon targeting and muscle spindle differentiation. Presently we cannot completely rule out this possibility. However, no defects in axon pathfinding along the monosynaptic reflex arc or in muscle spindle differentiation have been noted in PV KO mice, which develop normally and show no apparent changes in their behavior or physical activity (Schwaller et al. 1999). These observations suggest that axonal targeting defects in *Bax/NT-3* double KO mice cannot be simply due to lack of PV expression in proprioceptive cells.

Our present results suggest that NT-3 acts as a short-range axon guidance cue for proprioceptive axons centrally and peripherally, as they navigate to their targets using other axon guidance cues. In its absence, these axons terminate in inappropriate loci. However, NT-3 may not be the only molecule that plays a role in targeting and terminal branching of sensory axons in the ventral spinal cord. NT-3 most likely acts cooperatively with other axon guidance molecules or by regulating expression of yet to be identified proprioceptive neuron-specific receptors/ligands for numerous axon guidance cues.

## Materials and Methods

### 

#### Generation of double KOs.

We crossed *Bax* KO females on a C57BL/6 background (Jackson Laboratory, Bar Harbor, Maine, United States) to *NT-3* heterozygote males on a 129 Sv background to generate double heterozygote animals. Progeny was genotyped with PCR, and animals heterozygous for both genes were bred to obtain the double KOs. Primers used for the *Bax* locus were R661, GTT GAC CAG AGT GGC GTA GG; R662, CCG CTT CCA TTG CTC AGC GG; and R663, GAG CTG ATC AGA ACC ATC ATG. Primers used for the *NT-3* locus were NT3A, CGT GGT GAG GTT CTA TTG GCT AC; NT3B, CAG AGC ACC CTG CCC AAA GCA GAG; NT3R, CCT TGA CAA TAC TGA ATG CC; and NEOF, GGG AAC TTC CTG ACT AGG. WT, *Bax* KO, and *NT-3* KO littermates were used as controls. A total of 11 *Bax/NT-3* double KO mice were analyzed, of these nine were P0 pups. The p75 colony on a 129 S1 background was received from Jackson Laboratory. We used tail DNA to genotype animals using the primers IMR0013, CTT GGG TGG AGA GGC TAT TC; IMR0014, AGG TGA GAT GAC AGG AGA TC (generic neo primers); IMR0710, TGT TAC GTT CTC TGA CGT GGT GAG; and IMR0711, TCA GCC CAG GGT GTG CAC TC (*p75* locus). For embryonic experiments, day of plug positivity was considered E0. All of the protocols used in this study were approved by the Louisiana State University Health Sciences Center Institutional Animal Care and Use Committee and conformed to the National Institutes of Health guidelines for use of experimental animals.

#### TrkA/TrkC immunohistochemistry.

Frozen spinal cord sections (10 μm thick) were blocked and incubated in a cocktail of rabbit anti-TrkA and goat anti-TrkC antibodies (gift of Dr. Reichardt; Huang et al. 1999), followed by a cocktail of CY3 conjugated donkey anti-rabbit and FITC conjugated donkey anti-goat antibodies (Chemicon, Temecula, California, United States) in the presence of 0.3% TritonX-100 and 10% normal donkey serum. For PV immunohistochemistry, sections were reacted with monoclonal mouse anti-PV antibody (Sigma, St. Louis, Missouri, United States), and developed by Vector fluorescein mouse on mouse kit (Vector Laboratories, Burlingame, California, United States). For quantification purposes, TrkA- and TrkC-labeled cells from four different DRGs of each genotype and age studied were counted, and ratios plotted.

#### Muscle spindle detection.

Gastrocnemius muscle was dissected out in P0 pups and sectioned longitudinally, or, in some cases, the whole leg at the level of gastrocnemius muscle with tibial nerve was sectioned in cross section. Sections were incubated with monoclonal S46 antibody (gift of Dr. Stockdale) reactive to the spindle-specific slow-tonic myosin heavy chain isoform and developed by Vector mouse on mouse kit as described above, followed by rabbit anti-NF-M antibody (Chemicon) and CY3 conjugated goat anti-rabbit antibody (Chemicon) in a sequential double-labeling protocol. In another method, DiI crystals were placed in DRGs of E17 embryos from different genotypes (*n =* 2), and the gastrocnemius muscle was isolated and sectioned into 40-μm-thick slices on a vibratome.

#### DiI labeling.

Spinal cords were dissected out with the DRGs attached, motor root was cut to prevent backfilling of motor neurons, and crystals of 1,1′-dioctadecyl-3,3,3′,3′-tetramethylindocarbocyanine perchlorate (DiI; Molecular Probes, Eugene, Oregon, United States) were inserted in DRGs. Labeled spinal cords were incubated at 37 °C for 8 d and sectioned on a vibrotome at 100 μm. Sections were observed under the fluorescent microscope and photographed, and images were transferred to Adobe Photoshop, inverted, and adjusted for brightness and contrast. Photoconverted in 0.15% DAB (3,3′-diaminobenzidine; Sigma) in 0.1M Tris buffer (pH 8.2).

#### In vitro co-culture assay.

DRG explants were derived from Swiss Webster, Bax null, and p75 null mouse embryos (E13). For collagen matrix assays, DRGs were dissected out under a stereomicroscope using tungsten needles. Collagen matrix was prepared with 430 μl of collagen (3 mg/ml dissolved in 0.1M acetic acid, Sigma), 50 μl of 10X DMEM medium, and 2.5 μl of 0.8 M NaCa_2_, and the pH was adjusted to 7.5. Individual ganglion explants were placed in 24-well plates and covered with freshly prepared collagen. Sepharose beads with an average diameter of 150 μm were used. Beads were washed twice with PBS, air dried, and loaded with 10, 20, 50, or 100 ng/μl NT-3 (Collaborative Research, Chemicon) at 4 °C overnight with constant shaking. For negative control, beads were loaded with BSA (10–100 ng/μl) or PBS. Either a single neurotrophin-loaded bead or a single BSA (or PBS)–loaded bead was implanted about 200–1,200 μm away from the ganglion explant. Collagen-embedded cultures were then placed at 33 °C for 15 min for the matrix to harden. Serum-free culture medium was then added to each well. In cultures with WT DRG and control beads, 10% serum was added to ensure viability of the explants. TrkC-Fc (Regeneron Pharmaceuticals, Tarrytown, New York, United States) was added (20 μg/ml) into the culture medium.

#### Explant co-cultures

Spinal cords with attached DRGs were dissected out from Swiss Webster mouse embryos at E13, and sectioned into 300-μm-thick slices. Tissue slices were placed on Millicell Tissue Culture Inserts (Millipore, Billrica, Massachusetts, United States). NT-3-loaded (*n =* 15) or PBS-loaded (*n =* 6) sepharose beads were prepared as described above, and placed in the midline at mid-spinal cord level. Inserts were placed in six-well plates with serum-free medium at the bottom of the wells, and kept at 33 °C for 3 d in the presence of 5% CO_2_. Cultures were then fixed with 4% paraformaldehyde in PBS, and small crystals of DiI were placed into the DRGs. Samples were incubated in a 37-°C incubator, allowing the dye to diffuse, and photographed under a Nikon (Tokyo, Japan) inverted epifluoresence microscope.

## Supporting Information

Figure S1 Motor Neuron Innervation Initiated in WT E17 Embryos Cannot Be Detected in *Bax/NT-3* Double KOs In some of the samples, motor neurons were labeled by backfilling through the ventral root in addition to the sensory axons labeled through the DRGs.
(A) WT embryo showing proprioceptive axons contacting motor neuron dendrites in the ventral horn, forming synapses.
(B) Bax/NT-3 double null spinal cord. Although labeled fibers enter the ventral spinal cord, they extend towards the midline instead of the ventral horn and never contact the motor neuron dendrites.
(C) High-power image of the inset in (A). Arrows point to the proprioceptive fibers contacting motor neurons (asterisk).
(D) High-power image of the inset in (B). Notice that there are no sensory axons contacting labeled motor neurons (asterisk).
Scale bar: 100 μm (A and B), 50 μm (C and D).
(24 MB TIF).

Figure S2 S46/NF-M Immunohistochemistry at E15 Gastrocnemius Muscle Although numerous muscle and nerve fibers were labeled, no muscle spindles could be identified because the characteristic morphology of sensory nerve ending wrapped around muscle bag fiber had not begun to develop in any of the genotypes yet. Scale bar: 25 μm.
(13 MB TIF).

## Abbreviations

BDNF: brain-derived neurotrophic factor
BSA: bovine serum albumin
DRG: dorsal root ganglion
E: embryonic day
KO: knockout
NF-M: neurofilament-M
NT-3: neurotrophin-3
P: postnatal day
PBS: phosphate buffer saline
PV: parvalbumin
TrkC-Fc: diffusible TrkC receptors conjugated to IgG constant regions
WT: wild-type
