# Supplementary figures and images for: A Chemoattractant Role for NT-3 in Proprioceptive Axon Guidance
Source: PLoS Biol. 2004 Nov 23;2(12):e403. doi: 10.1371/journal.pbio.0020403 (PMC529315; doi:10.1371/journal.pbio.0020403)

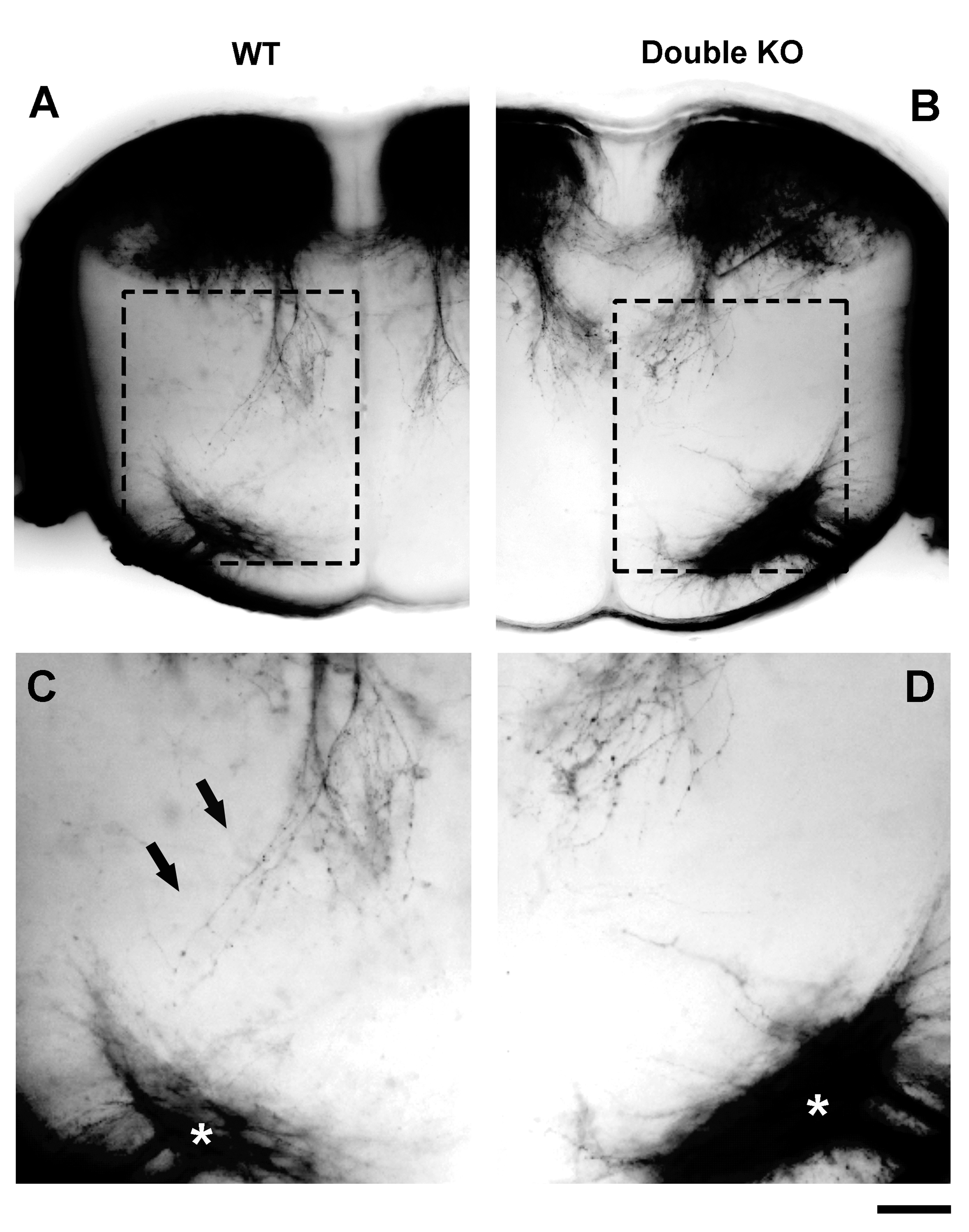

Supplement: Figure S1 — In some of the samples, motor neurons were labeled by backfilling through the ventral root in addition to the sensory axons labeled through the DRGs. (A) WT embryo showing proprioceptive axons contacting motor neuron dendrites in the ventral horn, forming synapses. (B) Bax/NT-3 double null spinal cord. Although labeled fibers enter the ventral spinal cord, they extend towards the midline instead of the ventral horn and never contact the motor neuron dendrites. (C) High-power image of the inset in (A). Arrows point to the proprioceptive fibers contacting motor neurons (asterisk). (D) High-power image of the inset in (B). Notice that there are no sensory axons contacting labeled motor neurons (asterisk). Scale bar: 100 μm (A and B), 50 μm (C and D). (24 MB TIF). [file pbio.0020403.sg001.tif]

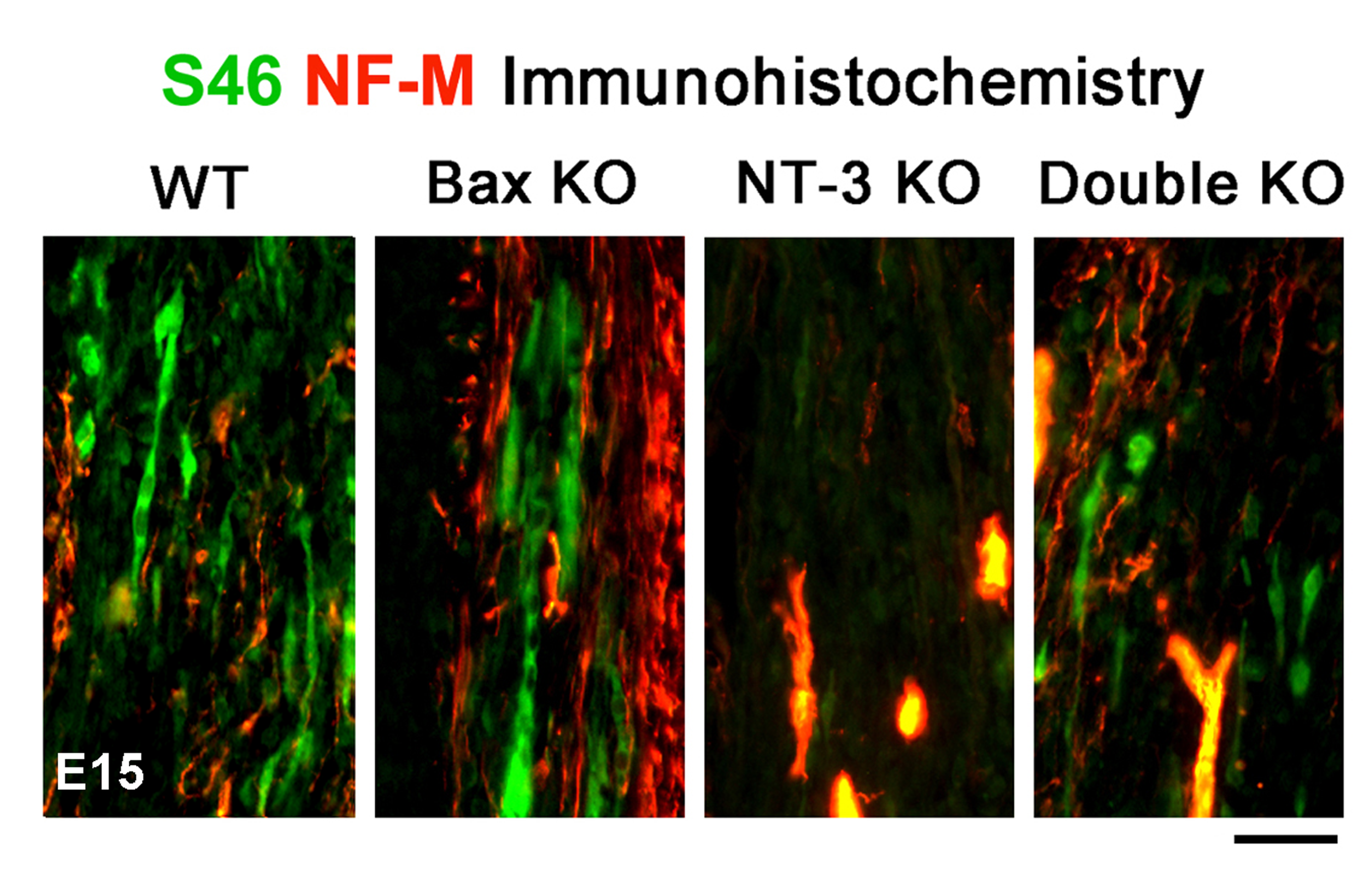

Supplement: Figure S2 — Although numerous muscle and nerve fibers were labeled, no muscle spindles could be identified because the characteristic morphology of sensory nerve ending wrapped around muscle bag fiber had not begun to develop in any of the genotypes yet. Scale bar: 25 μm. (13 MB TIF). [file pbio.0020403.sg002.tif]
